# Effects of auricular acupressure on chemotherapy-induced nausea and vomiting in breast cancer patients: a preliminary randomized controlled trial

**DOI:** 10.1186/s12906-022-03543-y

**Published:** 2022-03-24

**Authors:** Jing-Yu Tan, Alex Molassiotis, Lorna K. P. Suen, Jian Liu, Tao Wang, Hui-Rong Huang

**Affiliations:** 1 School of Nursing, The Hong Kong Polytechnic University, Hung Hom, Kowloon, Hong Kong SAR; 2 College of Nursing and Midwifery, Charles Darwin University, Casuarina Campus, Ellengowan Drive, Brinkin, NT 0810 Australia; 3 School of Nursing, Tung Wah College, Homantin Kowloon, Hong Kong SAR; 4 Department of Breast Oncology, Fujian Provincial Cancer Hospital, Fuma Road, Jinan District, Fuzhou, 340014 Fujian China; 5 The Second Affiliated Hospital of Fujian University of Traditional Chinese Medicine, Wusi Road, Gulou District, Fuzhou, 350003 Fujian China; 6 The Affiliated People’s Hospital of Fujian University of Traditional Chinese Medicine, 817 Mid Road, Taijiang District, Fuzhou, 350004 Fujian China

**Keywords:** Auricular therapy, Nausea and vomiting, Chemotherapy, Neoplasms, Randomized controlled trial

## Abstract

**Background:**

Auricular acupressure (AA) has been viewed as a promising approach to managing chemotherapy-induced nausea and vomiting (CINV) but relevant research evidence has been inconclusive. This study aimed to examine the effects of AA on CINV in breast cancer (BC) patients undergoing chemotherapy.

**Methods:**

A preliminary randomized controlled trial was conducted in 114 BC patients. Participants were randomly allocated to a true AA group (*n* = 38), a sham AA group (*n* = 38), and a standard care group (*n* = 38). All the participants were provided with standard antiemetic treatment and care, while the true AA group and the sham AA group received an additional 5-day true AA and a 5-day sham AA, respectively. Acute and delayed CINV were assessed by using the MASCC Antiemesis Tool (MAT), anticipatory nausea and vomiting were measured by the Index of Nausea, Vomiting, and Retching (INVR), and patients’ quality of life (QoL) was evaluated by the Functional Assessment of Cancer Therapy-Breast (FACT-B).

**Results:**

Both the true and sham AA groups reported improved CINV outcomes than the standard care group, with the true AA demonstrating larger effects than the sham comparison. The true and sham AA groups had higher complete response (CR) rates of CINV when compared with the standard care group, with the difference in the CR of acute CINV achieving statistical significance (*p* = 0.03). Both the true and sham AA groups demonstrated lower incidence and severity of acute CINV compared with the standard care group with the among-group difference reaching statistical significance for the occurrence (*p* = 0.04) and severity (*p* = 0.001) of acute nausea. No significant differences in anticipatory CINV and QoL were found among the groups.

**Conclusion:**

The use of AA plus standard antiemetic treatment and care was superior to the use of standard antiemetic treatment and care alone in managing CINV among BC patients receiving chemotherapy. The antiemetic effects of AA were identified to be more profound in improving acute CINV, particularly acute nausea. The antiemetic effects of AA were deemed to be a mixture of specific treatment effects and placebo effects, and the placebo effects were very large and even reached clinical significance.

**Trial registration:**

ClinicalTrials.gov; NCT02403037; Registered March 31, 2015.

## Background

Chemotherapy has tremendously improved cancer patients’ survival rates and long-term prognosis. However, chemotherapeutic regimens also lead to various side effects and symptoms that can significantly impede patients’ physical and psychosocial well-being. Nausea and vomiting are two frequently experienced symptoms during chemotherapy and are generally referred to as chemotherapy-induced nausea and vomiting (CINV) [1]. Prophylaxis and treatment of CINV have been challenging even with the use of antiemetics. Prospective observational studies have shown that, among cancer patients receiving moderately to highly emetogenic chemotherapy, the incidence of acute and delayed vomiting ranged from 8.3% to 24.5%, and from 16.5% to 29.5%, respectively, while the prevalence of nausea can be as double even triple as the incidence of vomiting, with the incidence of acute nausea and delayed nausea reported to be 23.3% to 46.0%, and 35.0% to 82.7%, respectively [2–6]. Delayed CINV symptoms have been more frequently identified than acute ones, and the management of nausea has been more problematic than vomiting [2–7]. Uncontrolled CINV can contribute to various medical complications, lead to impaired functioning status and quality of life (QoL), and increase patients’ physical, financial, and chemical burden [8–11]. All of which can significantly impede patients’ adherence to routine cancer treatments and further impact their long-term survival. Female gender and the use of highly emetogenic chemotherapeutic agents are universally regarded as risk factors for CINV [12–16], which make breast cancer (BC) patients one of the most vulnerable groups for CINV [17, 18].

The use of antiemetics following international antiemetic guidelines has been regarded as the most effective method for the prophylaxis and treatment of CINV [19]. NK_1_ receptor antagonists and 5-HT_3_ receptor antagonists have been recommended as the first-line treatment for CINV [19]. However, CINV symptoms are difficult to be completely managed by using antiemetics alone, and other complementary health interventions have therefore been recommended for use in combination with antiemetics for comprehensive management of CINV [20]. Auricular therapy as a popular complementary health approach has been commonly adopted for managing various health conditions [21–24]. The underpinning theories of auricular therapy recognise that health disorders occurred in certain body parts have their specific projections to sensitive areas and points in the outer auricle (the “auricular acupoints”), while auricular therapy practitioners can use different invasive or non-invasive approaches such as acupuncture and acupressure to stimulate such acupoints to alleviate the targeted pathological conditions [25, 26]. Auricular therapy has been utilized for CINV management, and one of our previous systematic reviews identified a few small-scale studies published during the past decade [27]. However, research evidence regarding the effects of auricular therapy on CINV remains inconclusive given that significant methodological flaws were identified in existing studies with various degrees of risk of bias, including a lack of detailed baseline assessment and blinding design, a failure of appropriate reporting of randomization and allocation concealment, and unsatisfactory outcome assessment without using reliable instruments for CINV, QoL and safety assessment [27]. Review findings highlighted that the development of existing auricular therapy interventions was not evidence-based, and auricular therapy protocols varied significantly across trials in terms of acupoint selection, intervention duration, and treatment sessions [27]. Placebo effects of auricular therapy for CINV management have also been uncertain given the lack of sham comparison utilized in published trials [27].

Our team has therefore conducted a research program to develop and evaluate an evidence-based auricular therapy intervention using non-invasive auricular acupressure (AA) approach to managing CINV [28]. In study phase I, an evidence-based AA intervention protocol was comprehensively developed based on evidence and recommendations concluded from systematic reviews, clinical trials and practice standards, AA-related theories, and expert panel consensus [29]. Study phase II included both a preliminary randomized controlled trial (RCT) and semi-structured interviews, where the antiemetic effects of AA were preliminarily evaluated in the RCT through a series of clinical outcome measures of CINV and QoL, while the feasibility assessment of the AA intervention was comprehensively evaluated across the RCT and the semi-structured interviews by using both quantitative and qualitative approaches [28]. The current paper presents the clinical outcomes of the RCT to preliminarily evaluate the effects of AA on CINV and QoL in 114 female BC patients undergoing the first cycle of chemotherapy.

## Methods

### Study design and settings

A three-arm sham-controlled RCT with a pilot study design (NCT02403037) was utilised. A partial blinding design of study intervention and outcome assessment was applied to participants in the true and sham intervention groups. Subject recruitment took place in three tertiary medical centres in Fuzhou, Fujian, China. This study adheres to CONSORT guidelines, and ethical clearance was granted by the Human Subjects Ethics Sub-Committee at The Hong Kong Polytechnic University, as well as the Clinical Trials Ethics Committees at the study sites. All participants were provided with verbal and printed information about this study, and all gave written informed consent prior to participating in this study.

### Study participants and sample size

Female adult BC patients were invited for study participation if they: (1) had a confirmed diagnosis of BC, stage I to III (without distant metastasis); (2) chemotherapy-naïve; (3) auricular therapy-naïve; (4) were able to communicate in Mandarin Chinese; (5) had at least primary school education; (6) agreed to participate in the study and were willing to provide written informed consent; (7) were scheduled to receive the first cycle of chemotherapy with moderately-high to highly emetogenic potential, such as anthracycline-based regimens including AC combination (doxorubicin plus cyclophosphamide), with or without paclitaxel, and EC combination (epirubicin plus cyclophosphamide), with or without paclitaxel/docetaxel; TC combination (cyclophosphamide plus docetaxel); and other less-frequently used combinations with moderately-high to highly emetogenic potential; and (8) were provided with standard antiemetics, including 5-HT3 receptor antagonists and/or dexamethasone.

Patients were excluded if they were: (1) extremely weak, disabled, or had immune deficiency; (2) were unable to follow the study instructions and cooperate with the study procedures; (3) had concurrent radiotherapy or other antineoplastic treatments; (4) were participating in other clinical studies; (5) had other health problems that may interfere with the CINV symptoms, such as gastrointestinal conditions, migraines, and tinnitus, etc.; (6) had ear skin problems that were not appropriate for AA.

For a pilot randomized trial, 30 to 50 participants per group would be sufficient for the parameter estimations for a future study [30]. Hertzog [31] also recommended that 30 subjects per group would be necessary for a pilot study with between-group comparison to perform effect size and confidence interval (CI) estimations for power analysis in future studies, which is in line with Browne’s suggestion (cited in [32]). Thirty participants per group were deemed as an appropriate sample size for this study. Considering a potential attrition rate of 20%, the final sample size was determined as 114, with 38 in each group.

### Study arms, randomization and blinding

Three groups, including a true AA group, a sham AA group, and a standard care group, were designed. Block randomization with a computer-based randomization sequence was utilized. Central randomization was adopted to ensure satisfactory allocation concealment. The randomization table was prepared by a university researcher (the “randomization person”) who was not involved in other study procedures. When an eligible participant was identified, the study investigators contacted the randomization person to request the corresponding group allocation.

Due to the visible nature of the AA intervention, a complete blinding design among all the three study groups was deemed impossible. A partial blinding design was therefore utilized in this study, where participants allocated to the true AA and sham AA groups, as well as the care providers, would not know whether the AA treatment was a true or sham method. A partial blinding design of outcome assessment for the true and sham AA groups was also maintained given that the clinical outcomes were all patient-reported scales and the participants themselves were deemed as the outcome assessors. Study investigators who were responsible for the subject recruitment and intervention delivery were aware of the group allocation.

### Study interventions

All the participants were provided with standard antiemetic treatment and care. 5-HT3 receptor antagonists and/or dexamethasone were administered prior to the commencement of chemotherapy, and the participants continued to receive prescribed antiemetics for the following one to two days post-chemotherapy. Daily care followed routine methods of care at the study sites. Those allocated to the true AA group and the sham AA group received an additional 5-day true AA, and an additional 5-day sham AA, respectively. The AA protocols, including both the true AA and sham AA, were developed by following the *Medical Research Council Framework for Developing and Evaluating Complex Intervention* [33], and based on AA-related theories, symptom characteristics of CINV, and evidence and recommendations concluded from relevant systematic reviews, clinical trials and practice standards [29]. Details of the development and validation of the AA intervention were separately reported in a methodological paper [29], and the true AA and sham AA protocols are briefly described as follows.

**Fig. 1:**
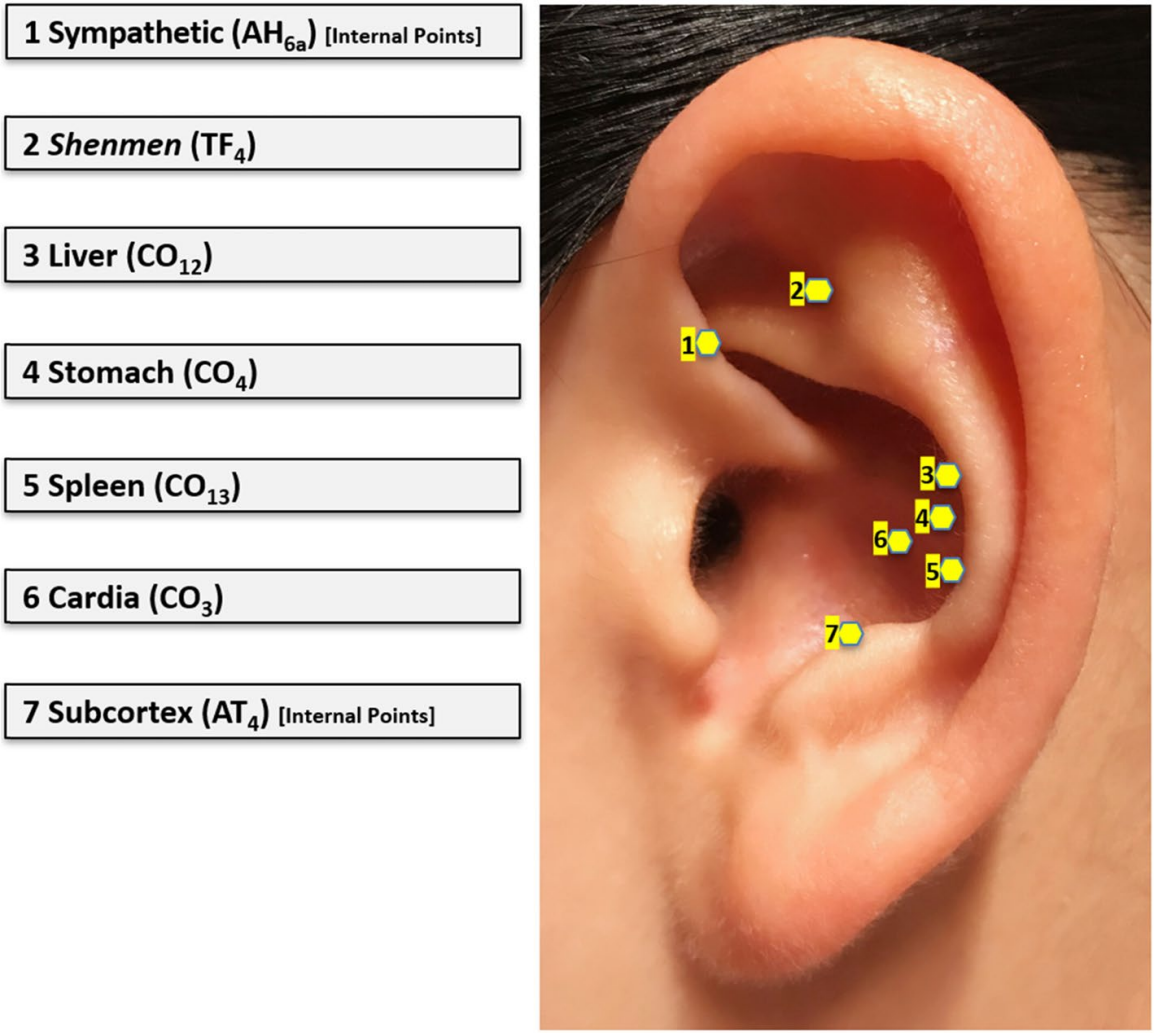
Selected acupoints in the AA intervention protocol for CINV management

The true AA was conducted from Day 1 to Day 5 of the first chemotherapy cycle. Seven acupoints that are closely related to the alleviation of emesis symptoms were selected, including “Cardia”, “Stomach”, “Spleen”, “Liver”, “*Shenmen*”, “Sympathetic”, and “Subcortex” (Fig. 1). An acupoint detector was used to locate the selected acupoints, and vaccaria seeds as the most commonly used medium for AA were attached to the acupoints using hypoallergenic tapes. Participants were instructed to press the taped seeds until achieving a sensation of heaviness, soreness, distension or tingling— the “*deqi*” sensation. AA was conducted three times daily in the morning, afternoon, and evening, with each time lasting about 4 to 7 min, adding pressure to all the seeds on both ears. In addition to the regular acupressure, the participants were told to do additional AA when they had the feeling of nausea.

The sham AA was performed from Day 1 to Day 5 of the first chemotherapy cycle, with the same acupoints and intervention duration utilised in the true AA to maintain a satisfactory blinding design among participants in the two AA groups. No manual acupressure was scheduled for the sham AA, and the vaccaria seeds used in the true AA were further replaced by Junci Medulla in the sham AA, which is very soft in texture to avoid the generation of “*deqi*” sensation (the specific treatment effects of AA) introduced by constant stimulation of the ear acupoints.

Subject recruitment and AA instruction were performed by three investigators who are registered nurses with AA training backgrounds. All the investigators were further trained by a senior researcher specialising in auricular therapy to standardise their AA practice in accordance with the AA intervention protocols. Verbal communication between the investigators and the participants, including an introduction of the AA procedure, and the instructions and precautions of self-acupressure, were standardized among the investigators.

### Study procedures

Potentially eligible patients were initially screened by the oncology nurses at the study sites. Potential participants who were interested in this study were referred to the investigators for further eligibility assessment. Eligible patients who agreed to participate were asked to complete an informed consent and baseline assessment before group assignment. Both the true and sham AA were delivered on Day 1 of the first chemotherapy cycle. For those receiving the true AA, the investigators first attached the vaccaria seeds to the selected acupoints and then demonstrated the self-acupressure technique. Participants were asked to perform a return demonstration to ensure that they had mastered the self-acupressure skills. The participants in the true AA group were provided with a log to record their daily AA practice and any possible AA-related adverse events. The participants in the sham AA group were given another type of daily log to record possible AA-related adverse events only. Participants’ adherence to the acupressure was monitored daily during the days when they stayed in the study hospitals; those who were discharged on Day 4 or Day 5 of the first chemotherapy cycle were reminded to continue with their daily AA and log recording. Telephone follow-up was conducted to further monitor their adherence to the study protocol.

**Fig. 2:**
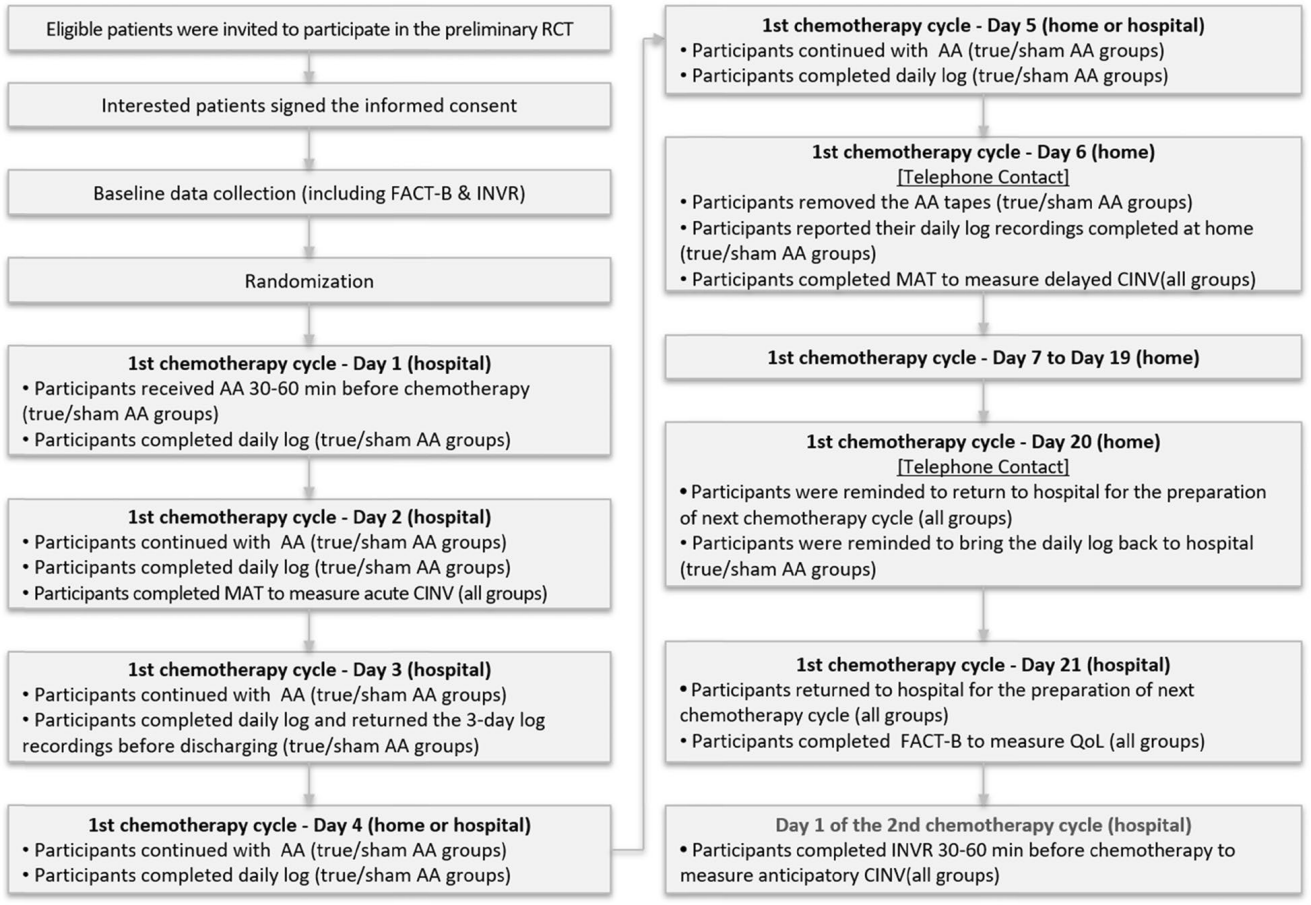
Methodological procedures of the preliminary RCT

Clinical outcomes were collected through patient-reported questionnaires, and two research assistants assisted with the data collection. Acute and delayed CINV symptoms were assessed on Day 2 and Day 6 of the first chemotherapy cycle, respectively, by using the MASCC Antiemesis Tool (MAT). Acute CINV was collected at the study sites while participants remained in the hospital, while the delayed CINV assessment was mostly conducted by telephone given that most of the participants were discharged on Day 4 or Day 5 of the first chemotherapy cycle. A short-term follow-up with two telephone calls was conducted after the completion of the AA treatment until the end of the first chemotherapy cycle (Day 21). Participants’ QoL was measured by using the Functional Assessment of Cancer Therapy-Breast (FACT-B) at baseline and the end of the first chemotherapy cycle (Day 21). Anticipatory nausea and vomiting were assessed by using the Index of Nausea, Vomiting and Retching (INVR) at baseline and on Day 1 of the second chemotherapy cycle before administering the chemotherapeutic agents (Fig. 2).

### Clinical outcome measures

Feasibility assessment of the AA intervention will be comprehensively analyzed, interpreted, and detailed in a separate paper that incorporates both the RCT feasibility outcomes and the nested semi-structured interview findings from both quantitative and qualitative perspectives. Clinical outcomes of the preliminary RCT, including acute and delayed CINV, anticipatory nausea and vomiting, QoL, and safety of AA, were detailed as follows.

#### Baseline assessment

The participants’ demographic data, medical history, CINV-related risk factors, and other baseline data were collected via a baseline assessment form. The participants’ QoL and anticipatory nausea and vomiting at baseline were also assessed using the FACT-B and the INVR, respectively.

#### Acute and delayed CINV

The MAT is the only available tool that separately records the acute and delayed CINV [34]. The MAT has eight items that assess the onset of vomiting, frequency of vomiting, onset of nausea, and intensity of nausea. Excellent psychometric properties of the Mandarin Chinese version were reported among a group of Chinese cancer patients with various cancer diagnoses [35]. Each MAT item can be assessed on its own, and a total CINV score, nausea score, vomiting score, and different domain scores (acute/delayed CINV, acute/delayed nausea, and acute/delayed vomiting) can be generated by summing up relevant items, with a lower score indicating a better outcome [34, 35].

#### Anticipatory nausea and vomiting

The INVR has eight items that contribute to three domains— symptom experience, symptom occurrence, and symptom distress. Each item is rated on a 5-point Likert scale (0–4), and the total score can range from 0 to 32, with a higher score indicating a more severe emesis outcome [36–38]. Psychometric properties of the INVR Mandarin Chinese version have been well proved in Chinese cancer and obstetric patients [39].

#### QoL

The FACT-B is a BC-specific QoL assessment that consists of four general subscales (physical, social/family, emotional, and functional well-being) and one BC-specific subscale [40, 41]. Each item of the FACT-B is rated on a 5-point Likert scale (0–4), and the total score can range from 0 to 148, with a higher score reflecting a better QoL [42, 43]. The FACT-B Mandarin Chinese version was utilized in this study, with appropriate psychometric properties documented among Chinese BC patients [43].

#### Safety of AA

AA-related adverse events were recorded and reported by the participants. The likelihood of causality between the adverse reactions and the AA was determined by a senior acupuncturist. The *World Health Organization-Uppsala Monitoring Centre System for Standardized Case Causality Assessment* was adapted to identify the causality between the AA and the adverse events, with six degrees of causality to classify the likelihood: “certain,” “probable/likely,” “possible,” “unlikely,” “conditional/unclassified,” and “unassessable/unclassifiable” [44].

### Data analysis

The IBM SPSS Statistics for Windows 23.0 was utilized for data analysis with the significance level set as *p* < 0.05 for a two-tailed test. Data analysis followed the principle of intention-to-treat (ITT) analysis, and missing data were handled with the last observation carried forward (LOCF) approach. The Chi-square test/Fisher’s exact test and the one-way ANOVA/Welch ANOVA/Kruskal–Wallis test was utilized to perform the baseline comparisons based on the types of data and the results for normality and homogeneity of variance.

A complete response (CR) of CINV symptoms and the incidence of nausea and vomiting were estimated based on the MAT single items, with the Chi-square test/Fisher’s exact test performed for among-group comparisons followed by a post-hoc test with partitioning Chi-squared statistics for comparisons showing statistical significance. A 10% change in the CR of CINV symptoms or incidence of nausea and vomiting between groups was utilized to indicate the clinical significance of the change in CINV symptoms [45–47].

For the frequency of acute/delayed vomiting, the severity of acute/delayed nausea, as well as the MAT, INVR, and FACT-B scores, the one-way ANOVA/Welch ANOVA/Kruskal–Wallis test was conducted to identify the among-group differences, with a post-hoc analysis using the Tukey’s test/Games-Howell test/Dunn-Bonferroni test for comparisons showing statistical significance. The occurrence of anticipatory nausea and vomiting at baseline and post-intervention assessments was also recorded using the INVR if the participants scored any INVR item related to nausea or vomiting as “1” or above. Among-group differences were examined using the Chi-square test/Fisher’s exact test, with a post-hoc test with partitioning Chi-squared statistics for comparisons demonstrating statistical significance.

Effect sizes were estimated for relevant among-group comparisons. Cramer’s *V* was adopted for comparisons using the Chi-square test/Fisher’s exact test [48, 49]; *η2* was utilised for comparisons using the Kruskal–Wallis H test [50, 51]; while the partial *η2* was used for comparisons utilizing ANOVA [51, 52]. For Cramer’s *V* with a degree of freedom of 2, a value of 0.07 indicates a small effect size, 0.21 means a medium effect size, and 0.35 represents a large effect size [53, 54]; for *η*2, a value of 0.01, 0.06, and 0.14 represent a small, medium, and large effect size, respectively [53, 54]; effect size of the partial *η*2 was interpreted as the same as that of the *η*2 [55, 56]. Potential confounding effects introduced by the insignificant variations in participants’ baseline CINV risk factors were further addressed by using the generalized estimating equation (GEE) model as an additional analysis of this study.

## Results

### Subject recruitment and baseline characteristics

**Fig. 3:**
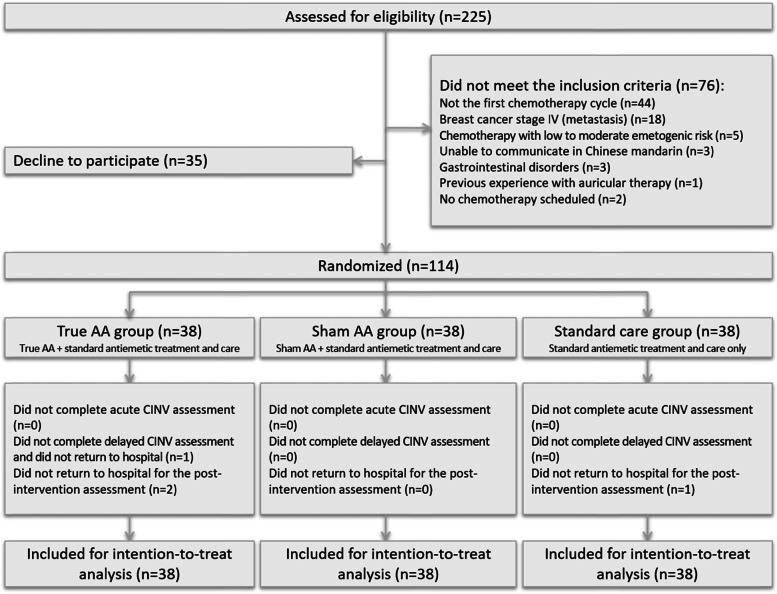
CONSORT diagram of the preliminary RCT procedure

Two hundred and twenty-five BC patients were screened for eligibility. One hundred and fourteen of them took part in the RCT and 110 completed the entire study procedure (Fig. 3). Subject recruitment was completed during a 15-month period. The eligibility rate for the screened patients was 66.2% (149/225), and the recruitment rate was 76.5% (114/149). The retention rate of the participants was 96.5% (110/114), which donated a minimal attrition rate of 3.5%. Participants’ baseline and clinical data are presented in Table 1 and they were generally comparable without any statistically significant difference across groups.

**Table 1:** Baseline demographic and clinical characteristics of the participants (*N* = 114)

**Variables**	**True AA** **(*n* = 38)** (%)	**Sham AA** **(*n* = 38)** (%)	**Standard Care** **(*n* = 38)** (%)	**Total** **(*n* = 114)** (%)	**Homogeneity Statistics** (*p*)
**Age (Mean ± SD)**	46.2 ± 7.1	47.9 ± 9.9	48.4 ± 9.3	47.5 ± 8.8	0.54
**Education background**					
Primary school	18 (47.4%)	19 (50.0%)	17 (44.7%)	54 (47.4%)	0.27
Secondary school	6 (15.8%)	10 (26.3%)	8 (21.1%)	24 (21.1%)	
High school/technical school	11 (28.9%)	7 (18.4%)	5 (13.2%)	23 (20.2%)	
College diploma/university degree	3 (7.9%)	2 (5.3%)	8 (21.1%)	13 (11.4%)	
**Marital status**					
Single	1 (2.6%)	0 (0.0%)	0 (0.0%)	1 (0.9%)	> 0.99
Married	37 (97.4%)	38 (100%)	38 (100%)	113 (99.1%)	
**Employment status**					
Professional	8 (21.1%)	5 (13.2%)	6 (15.8%)	19 (16.7%)	0.91
Manual work	8 (21.1%)	7 (18.4%)	7 (18.4%)	22 (19.3%)	
Housewife	17 (44.7%)	14 (36.8%)	16 (42.1%)	47 (41.2%)	
Admin/clerical	2 (5.3%)	4 (10.5%)	3 (7.9%)	9 (7.9%)	
Retired	1 (2.6%)	4 (10.5%)	4 (10.5%)	9 (7.9%)	
Other	2 (5.3%)	4 (10.5%)	2 (5.3%)	8 (7.0%)	
**Religious background**					
Buddhist	13 (34.2%)	14 (36.8%)	11 (28.9%)	38 (33.3%)	0.32
Christian	4 (10.5%)	0 (0.0%)	2 (5.3%)	6 (5.3%)	
None	21 (55.3%)	24 (63.2%)	25 (65.8%)	70 (61.4%)	
**Family monthly income**					
< 3000 CNY	6 (15.8%)	8 (21.1%)	10 (26.3%)	24 (21.1%)	0.22
3000–6000 CNY	15 (39.5%)	22 (57.9%)	16 (42.1%)	53 (46.5%)	
6001–10,000 CNY	13 (34.2%)	5 (13.2%)	11 (28.9%)	29 (25.4%)	
> 10,000 CNY	4 (10.5%)	3 (7.9%)	1 (2.6%)	8 (7.0%)	
**Medical insurance**					
Free government medical service	3 (7.9%)	1 (2.6%)	4 (10.5%)	8 (7.0%)	0.23
Social insurance	12 (31.6%)	14 (36.8%)	17 (44.7%)	43 (37.7%)	
New rural cooperative medical service	22 (57.9%)	17 (44.7%)	15 (39.5%)	54 (47.4%)	
Self-financed	1 (2.6%)	6 (15.8%)	2 (5.3%)	9 (7.9%)	
**Cancer Stage**					
Stage I	5 (13.2%)	5 (13.2%)	5 (13.2%)	15 (13.2%)	0.43
Stage II	19 (50.0%)	26 (68.4%)	20 (52.6%)	65 (57.0%)	
Stage III	14 (36.8%)	7 (18.4%)	13 (34.2%)	34 (29.8%)	
**Surgery**					
Modified radical mastectomy	24 (63.2%)	30 (78.9%)	27 (71.1%)	81 (71.1%)	0.50
Simple mastectomy	1 (2.6%)	2 (5.3%)	2 (5.3%)	5 (4.4%)	
Breast-conserving surgery	2 (5.3%)	0 (0.0%)	2 (5.3%)	4 (3.5%)	
Other	3 (7.9%)	1 (2.6%)	4 (10.5%)	8 (7.0%)	
NA	8 (21.1%)	5 (13.2%)	3 (7.9%)	16 (14.0%)	
**Chemotherapy combination**					
AC/AC-T combination	15 (39.5%)	14 (36.8%)	12 (31.6%)	41 (36.0%)	0.99
EC/EC-T/EC-D combination	20 (52.6%)	21 (55.3%)	22 (57.9%)	63 (55.3%)	
TC combination	2 (5.3%)	2 (5.3%)	2 (5.3%)	6 (5.3%)	
Other ^a^	1 (2.6%)	1 (2.6%)	2 (5.3%)	4 (3.5%)	
**Antiemetic medication**					
5-HT3 antagonists + dexamethasone	22 (57.9%)	23 (60.5%)	22 (57.9%)	67 (58.8%)	> 0.99
5-HT3 antagonists only	16 (42.1%)	15 (39.5%)	15 (39.5%)	46 (40.4%)	
Dexamethasone only	0 (0.0%)	0 (0.0%)	1 (2.6%)	1 (0.9%)	
**Risk factor for CINV**					
Aged less than 50 years old	25 (65.8%)	23 (60.5%)	19 (50.0%)	67 (58.8%)	0.36
History of morning sickness	26 (68.4%)	27 (71.1%)	19 (50.0%)	72 (63.2%)	0.12
History of motion sickness	18 (47.4%)	16 (42.1%)	13 (34.2%)	47 (41.2%)	0.50
History of labyrinthitis	1 (2.6%)	1 (2.6%)	1 (2.6%)	3 (2.6%)	> 0.99
**Number of risk factor for CINV**					
3 risk factors or above	11 (28.9%)	7 (18.4%)	7 (18.4%)	25 (21.9%)	0.44
Less than 3 risk factors	27 (71.1%)	31 (81.6%)	31 (81.6%)	89 (78.1%)	

This table is derived and modified from the original PhD thesis of Professor Jing-Yu (Benjamin) Tan [Tan, J. (2017). Effects of auricular acupressure on chemotherapy-induced nausea and vomiting in breast cancer patients: a preliminary randomized controlled trial. Doctoral dissertation, The Hong Kong Polytechnic University, Hong Kong.] [28]

*AA* auricular acupressure, *NA* not applicable, *SD* standard deviation, *CNY* Chinese Yuan

AC = doxorubicin + cyclophosphamide; T = paclitaxel; EC = epirubicin + cyclophosphamide; D = docetaxel; TC = cyclophosphamide + docetaxel; a = other less frequently used chemotherapy combinations with moderately-high to highly emetogenic potential, including pirarubicin plus cyclophosphamide combination and pirarubicin/epirubicin combined with other chemotherapeutic agents with low to moderate emetogenic risks, *CINV* chemotherapy-induced nausea and vomiting

### Acute and delayed CINV symptoms

#### CR of CINV symptoms

The true AA group and the sham AA group had higher CR rates of all the CINV symptoms compared with the standard care group, while the CR rates between the true AA and the sham AA were generally similar. A statistically significant difference in the CR of acute CINV was identified among the three groups, with a medium effect size (*p* = 0.03, Cramer’s *V* = 0.25); the further post-hoc comparisons revealed a statistically significant difference in the CR of acute CINV between the true AA group and the standard care group (*p* = 0.01), and no statistical difference was identified between the true AA and the sham AA. A borderline significant difference was found between the sham AA group and the standard care group (*p* = 0.05) (Table 2). The differences in the CR of overall CINV, CR of acute CINV, CR of overall nausea, CR of overall vomiting reached clinical significance between the true AA group and the standard care group, and between the sham AA group and the standard care group.

**Table 2:** Differences in the complete response of CINV symptoms among the study groups

MAT Outcomes ^a^	True AA		Sham AA		Standard Care		Chi-square Test		Effect Size	Post-hoc Analysis ^c^					
										**True vs. Sham**		**True vs. SC**		**Sham vs. SC**	
	n ^b^	No. of CR (%)	n	No. of CR (%)	n	No. of CR (%)	Value	*p*	Cramer’s V	Value	*p*	Value	*p*	Value	*p*
**CR of overall CINV**	37	11 (29.7%)	38	11 (28.9%)	38	5 (13.2%)	3.64	0.16	0.18	NA	NA	NA	NA	NA	NA
**CR of acute CINV**	38	20 (52.6%)	38	17 (44.7%)	38	9 (23.7%)	7.07	**0.03**	0.25	0.47	0.49	6.75	**0.01**	3.74	0.05
**CR of delayed CINV**	37	16 (43.2%)	38	16 (42.1%)	38	13 (34.2%)	0.76	0.68	0.08	NA	NA	NA	NA	NA	NA
**CR of overall nausea**	37	11 (29.7%)	38	11 (28.9%)	38	5 (13.2%)	3.64	0.16	0.18	NA	NA	NA	NA	NA	NA
**CR of overall vomiting**	37	24 (64.9%)	38	24 (63.2%)	38	17 (44.7%)	3.85	0.15	0.19	NA	NA	NA	NA	NA	NA

This table is derived and modified from the original PhD thesis of Professor Jing-Yu (Benjamin) Tan [Tan, J. (2017). Effects of auricular acupressure on chemotherapy-induced nausea and vomiting in breast cancer patients: a preliminary randomized controlled trial. Doctoral dissertation, The Hong Kong Polytechnic University, Hong Kong.] [28]

*CINV* chemotherapy-induced nausea and vomiting, *MAT* MASCC Antiemesis Tool, *AA* auricular acupressure, *SC* standard care, *CR* complete response, *NA* not applicable

^a^CR of overall CINV: no nausea and vomiting from day 1 to day 5 of the first chemotherapy cycle; CR of acute CINV: no nausea and vomiting during day 1 of the first chemotherapy cycle; CR of delayed CINV: no nausea and vomiting from day 2 to day 5 of the first chemotherapy cycle; CR of overall nausea: no nausea from day 1 to day 5 of the chemotherapy cycle; CR of overall vomiting: no vomiting from day 1 to day 5 of the chemotherapy cycle

^b^one participant from the true AT group dropped out during the delayed CINV assessment. ITT analysis using the acute phase data as the delayed outcomes is not appropriate here because the delayed CINV symptom is different from the acute CINV symptom in nature. No ITT approach was utilized in this analysis; therefore, the available sample size for calculating the CR of the overall CINV, delayed CINV, overall nausea, and overall vomiting in the true AT group was 37

^c^partitioning Chi-square statistics were applied for the post-hoc analysis

#### Occurrence and severity of acute and delayed CINV

**Fig. 4:**
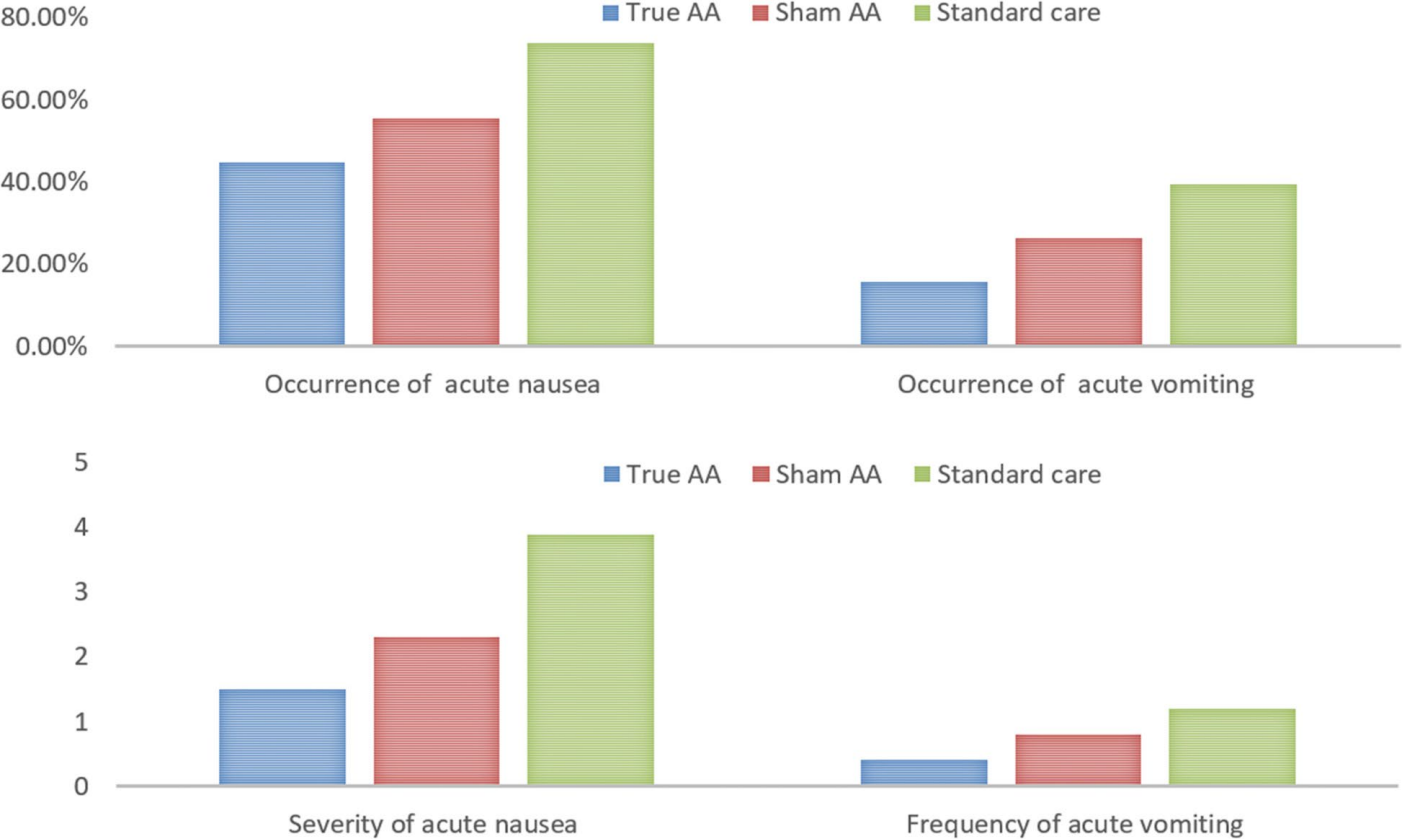
Occurrence, severity and frequency of acute CINV symptoms

The occurrence of acute nausea in the true AA group was lower than in the sham AA group, and both were lower than in the standard care group; a statistically significant difference was identified among the three groups, with a medium effect size (*p* = 0.04, Cramer’s *V* = 0.24). However, the following post-hoc tests showed only a statistically significant difference between the true AA group and the standard care group (*p* = 0.01). For the occurrence of acute vomiting, the true AA also demonstrated a lower incidence compared with the sham AA; both groups reported less acute vomiting than the standard care group; a borderline significant difference was found among the three groups with a medium effect (*p* = 0.07, Cramer’s *V* = 0.22). The mean score for the severity of acute nausea in the true AA group was much lower than in the sham AA group and the standard care group, with a statistically significant among-group difference and a near large effect size (*p* = 0.001, *η*2 = 0.13); further post-hoc comparisons revealed a statistically significant difference between the true AA group and the standard care group and a borderline significant difference between the sham AA group and the standard care group. The frequency of acute vomiting was also less in the true AA than in the sham AA when compared with the standard care group; a borderline significant difference was found among the three groups with a near medium effect size (*p* = 0.07, *η*2 = 0.05) (Fig. 4).

**Fig. 5:**
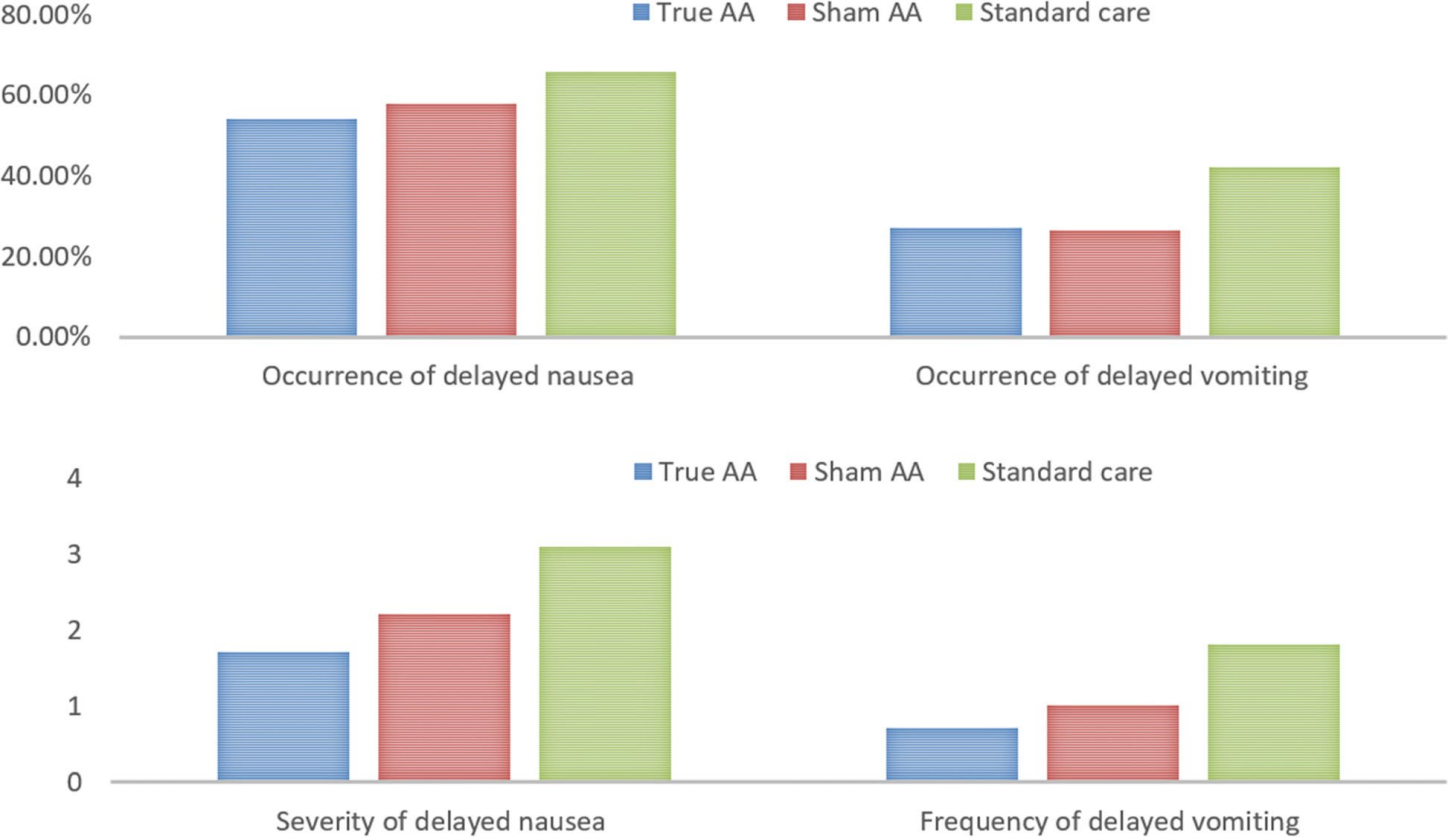
Occurrence, severity and frequency of delayed CINV symptoms

The occurrence of delayed nausea in the true AA was lower than in the sham AA, and both reported a relatively lower incidence than the standard care group, but there was no statistically significant difference found among groups. The severity score of delayed nausea in the true AA was lower than in the sham AA, and both reported a lower score than the standard care group. However, no statistical difference can be found among the three groups. Similarly, either the true AA group or the sham AA group showed a relatively lower frequency of delayed vomiting than the standard care group, without any among-group statistical differences (Fig. 5).

The differences in the occurrence of acute nausea, the occurrence of acute vomiting, and occurrence of delayed vomiting reached clinical significance between the true AA group and the standard care group, and between the sham AA group and the standard care group. Differences in the occurrence of delayed nausea reached clinical significance between the true AA group and the standard care group.

#### MAT total and domain scores

The true AA group demonstrated lower MAT total and domain scores when compared with the sham AA group; the scores in the sham AA group were also lower than in the standard care group; statistically significant differences in the MAT overall total (*p* = 0.004, *η*2 = 0.10), MAT total nausea (*p* = 0.003, *η*2 = 0.11), MAT acute CINV (*p* = 0.002, *η*2 = 0.11), and MAT acute nausea (*p* = 0.001, *η*2 = 0.13) were identified among the three groups with near large effect sizes; the post-hoc tests indicated statistically significant differences in the MAT overall total, MAT total nausea, MAT acute CINV, and MAT acute nausea between the true AA group and the standard care group, while no significant differences were detected between the true AA group and the sham AA group, and between the sham AA group and the standard care group (Tables 3 and 4).

**Table 3:** Differences in the MAT total and domain scores among the study groups

MAT Outcomes ^a^	True AA		Sham AA		Standard Care		Kruskal–Wallis H Test		Effect Size
	n ^b^	Mean/SD/SE/Median [95% CI]	n	Mean/SD/SE/Median [95% CI]	n	Mean/SD/SE/Median [95% CI]	Value	*p*	*η*^2^
**MAT overall total**	37	5.8/5.5/0.9/4.0 [3.9–7.6]	38	7.9/7.3/1.2/5.5 [5.5–10.4]	38	12.2/9.1/1.5/11 [9.2–15.2]	11.09	**0.004**	0.10
**MAT total nausea**	37	4.2/3.9/0.6/4.0 [2.9–5.5]	38	5.6/4.8/0.8/5.0 [4.1–7.2]	38	8.4/5.8/0.9/8.0 [6.6–10.3]	11.86	**0.003**	0.11
**MAT total vomiting**	37	1.6/2.6/0.4/0.0 [0.7–2.5]	38	2.3/3.6/0.6/0.0 [1.1–3.5]	38	3.8/4.8/0.8/2.0 [2.2–5.3]	4.47	0.11	0.04
**MAT acute CINV**	38	2.5/3.1/0.5/0.0 [1.5–3.5]	38	3.9/4.3/0.7/3.5 [2.5–5.4]	38	6.2/4.8/0.8/6.0 [4.7–7.8]	12.28	**0.002**	0.11
**MAT acute nausea**	38	1.9/2.3/0.4/0.0 [1.2–2.7]	38	2.9/2.9/0.5/3.5 [2.0–3.8]	38	4.7/3.5/0.6/5.0 [3.5–5.8]	14.38	**0.001**	0.13
**MAT acute vomiting**	38	0.6/1.4/0.2/0.0 [0.1–1.1]	38	1.1/2.0/0.3/0.0 [0.4–1.7]	38	1.6/2.2/0.4/0.0 [0.8–2.3]	5.44	0.07	0.05
**MAT delayed CINV**	37	3.2/3.6/0.6/2.0 [2.0–4.4]	38	4.0/4.7/0.8/3.0 [2.5–5.5]	38	6.0/5.8/0.9/5.0 [4.1–7.9]	4.27	0.12	0.04
**MAT delayed nausea**	37	2.2/2.5/0.4/2.0 [1.4–3.0]	38	2.7/2.8/0.5/3.0 [1.8–3.7]	38	3.8/3.4/0.6/3.5 [2.6–4.9]	4.23	0.12	0.04
**MAT delayed vomiting**	37	1.0/1.8/0.3/0.0 [0.4–1.6]	38	1.3/2.5/0.4/0.0 [0.4–2.1]	38	2.2/3.2/0.5/0.0 [1.2–3.3]	3.39	0.18	0.03

This table is derived and modified from the original PhD thesis of Professor Jing-Yu (Benjamin) Tan [Tan, J. (2017). Effects of auricular acupressure on chemotherapy-induced nausea and vomiting in breast cancer patients: a preliminary randomized controlled trial. Doctoral dissertation, The Hong Kong Polytechnic University, Hong Kong.] [28]

*MAT* MASCC Antiemesis Tool

^a^for MAT total scale and different symptom domains, a higher score indicates more severe nausea and/or vomiting; *AA* auricular acupressure

^b^one participant from the true AT group dropped out during the delayed CINV assessment. ITT analysis using the acute phase data as the delayed outcomes is not appropriate here because the delayed CINV symptom is different from acute CINV symptom in nature. No ITT approach was utilized in this analysis; therefore, the available sample size for computing the MAT overall total, MAT total nausea, MAT total vomiting, MAT delayed CINV, MAT delayed nausea, and MAT delayed vomiting scores in the true AT group was 37; *SD* standard deviation, *SE* standard error, *CI* confidence interval, *CINV* chemotherapy-induced nausea and vomiting

**Table 4:** Post-hoc analysis (Dunn-Bonferroni test) for MAT overall total, MAT total nausea, MAT acute CINV, and MAT acute nausea scores

MAT Outcomes ^a^	Post-hoc Analysis (Dunn-Bonferroni Test)					
	**True AA vs. Sham AA**		**True AA vs. Standard Care**		**Sham AA vs. Standard Care**	
	Statistic	Adjusted *p* value	Statistic	Adjusted *p* value	Statistic	Adjusted *p* value
**MAT overall total**	-8.66	0.75	-24.61	**0.003**	-15.95	0.10
**MAT total nausea**	-9.68	0.59	-25.56	**0.002**	-15.88	0.10
**MAT acute CINV**	-10.17	0.49	-25.47	**0.001**	-15.30	0.11
**MAT acute nausea**	-10.67	0.43	-27.34	**0.001**	-16.67	0.07

This table is derived and modified from the original PhD thesis of Professor Jing-Yu (Benjamin) Tan [Tan, J. (2017). Effects of auricular acupressure on chemotherapy-induced nausea and vomiting in breast cancer patients: a preliminary randomized controlled trial. Doctoral dissertation, The Hong Kong Polytechnic University, Hong Kong.] [28]

*MAT* MASCC Antiemesis Tool

^a^for MAT total scale and different symptom domains, a higher score indicates more severe nausea and/or vomiting; *AA* auricular acupressure; *CINV* chemotherapy-induced nausea and vomiting

### Anticipatory nausea and vomiting

Very few participants (three out of 114, one from each group) experienced anticipatory nausea before the first chemotherapy cycle, and no participants reported anticipatory vomiting. Nine reported anticipatory nausea before the second chemotherapy cycle, of which, four were from the true AA group, two were from the sham AA group, and three were from the standard care group. There was no statistically significant difference in the occurrence of anticipatory nausea among the three groups. Three participants (one from each group) experienced anticipatory vomiting prior to the second chemotherapy cycle.

### QoL

The post-intervention FACT-B change scores from baseline were utilised with a larger positive change indicating a better outcome. Relatively lower scores were identified in the majority of the FACT-B domains at post-intervention assessment than at baseline, which indicated deteriorated QoL after the first chemotherapy cycle. No statistically significant difference was found in the FACT-B total and domain scores among the three groups.

### Safety of the AA intervention

Table 5 summarises the reported AA-related side reactions. Eleven participants indicated minor to moderate adverse effects associated with AA, and the causalities between the AA treatment and the suspected adverse events were deemed as “probable/likely.” No serious adverse events occurred, and the reported adverse events were generally tolerable and transient. Participants also reported that adverse reactions gradually disappeared after removing the AA tapes without the need for additional intervention to manage the reported adverse events.

**Table 5:** Summary of AA-related adverse events in the true and placebo AA groups

**Type of AA-related AE**	**True AA (*n* = 38)**^**a**^ Number (%)	**Placebo AA (*n* = 38)** Number (%)	**Total (*n* = 76)** Number (%)	**Causality between AA and AE**^**b**^
Minor itching	0 (0.0%)	2 (5.3%)	2 (2.6%)	Probable/likely
Minor discomfort	2 (5.3%)	0 (0.0%)	2 (2.6%)	Probable/likely
Minor pain	5 (13.2%)	0 (0.0%)	5 (6.6%)	Probable/likely
Moderate pain	1 (2.6%)	0 (0.0%)	1 (1.3%)	Probable/likely
Minor to moderate pain	1 (2.6%)	0 (0.0%)	1 (1.3%)	Probable/likely

This table is derived and modified from the original PhD thesis of Professor Jing-Yu (Benjamin) Tan [Tan, J. (2017). Effects of auricular acupressure on chemotherapy-induced nausea and vomiting in breast cancer patients: a preliminary randomized controlled trial. Doctoral dissertation, The Hong Kong Polytechnic University, Hong Kong.] [28]

*AA* auricular acupressure, *AE* adverse events

^a^one of the three true AA group participants who dropped out of the preliminary RCT completed the five-day AA and provided via telephone her five-day AA daily log recordings that she completed at home, while the other two participants completed only a two-day AA and log recordings and a three-day AA and log recordings, respectively;

^b^causality between AA and the reported AE was determined using the *WHO-UMC System for Standardized Case Causality Assessment*

### Additional analysis for potential confounding effects at baseline

Although the participants’ baseline characteristics were comparable across groups, the CINV risk factors— younger age, history of morning sickness, and history of motion sickness, still showed some insignificant variations across groups. An additional analysis using the GEE model was therefore performed to explore the potential confounding effects of baseline CINV risk factors on the causality analysis between AA and CINV. The three factors were introduced one-by-one as a covariate into the GEE model, and the covariate-adjusted between-group mean differences and effect sizes for the MAT total and domain scores were then compared with the corresponding statistics in the covariate-unadjusted GEE model to examine whether there were any significant variations in those statistics before and after controlling the potential confounding factor.

The changes in the mean differences and related effect sizes in the majority of the between-group comparisons of the MAT scores were slightly larger in the covariate-adjusted GEE than in the covariate-unadjusted GEE. For instance, when adjusting the younger age factor, the mean difference in the MAT overall total score between the true AA and the sham AA was -2.2 (*p* = 0.14, Cohen’s *d* = 0.34) in the covariate-unadjusted GEE, while it was -2.3 (*p* = 0.11, Cohen’s *d* = 0.37) in the covariate-adjusted GEE. However, there were no obvious variations in those statistics before and after controlling for relevant risk factors, as the between-group analyses that showed significant (insignificant) differences in the covariate-unadjusted model continued to be significant (insignificant) in the covariate-adjusted model. Effect sizes for the between-group analyses that indicated small (medium or large) effects in the covariate-unadjusted model also continued to be small (medium or large) in the covariate-adjusted model.

## Discussion

This study revealed that the incidence of nausea was nearly twice higher than that of vomiting at both the acute and delayed CINV. The delayed vomiting was somewhat more frequently reported than the acute vomiting. These results are consistent with previous studies, that nausea is much more difficult to be controlled than vomiting, and delayed symptoms are also more common than acute ones [2–6]. This study demonstrated significant positive antiemetic effects of AA on acute nausea, which highlights the value of using AA as an adjuvant approach to antiemetics for CINV alleviation. The effects of AA on acute vomiting were also promising. However, this study did not identify any statistically significant positive effects of AA on delayed CINV, which partially reflects that delayed symptoms are more difficult to manage than acute ones. However, the study sample size was relatively small, and the sample size estimation was not power-based which indicates a risk of committing a type II error (an error of omission) in the data analysis. Nevertheless, the positive trend in the reduction of delayed CINV in the true AA and sham AA supports a potentially beneficial role of AA in managing delayed CINV. A future main study with a fully-powered sample size estimation is necessary to further evaluate the definite effects of AA on CINV.

The clinical significance of healthcare interventions can provide critical implications for clinical decision-making and health care policymaking [57]. Freiman et al. (cited in [58]) emphasized that insignificant findings merely concluded by statistical inference may have ignored the possibility that some of the statistically insignificant results might be clinically important. In this study, a 10% change in the CR of CINV symptoms or incidence of nausea and vomiting between groups was utilized to indicate the clinical significance of the change in CINV symptoms [45–47]. The results showed that the differences in the CR of overall CINV, acute CINV, overall nausea and overall vomiting, as well as the occurrence of acute nausea, acute vomiting, and delayed vomiting reached clinical significance between the true AA group and the standard care group, and between the sham AA group and the standard care group. Differences in the occurrence of delayed nausea also reached clinical importance between the true AA group and the standard care group. All of which indicates that the changes in the majority of CINV symptoms were clinically important to BC patients and clinicians, even for several outcomes showing insignificant changes in the statistical hypothesis testing. Considering both the statistical significance and clinical significance of the effects of AA on CINV, preliminary evidence can be concluded that AA can be utilized as an effective adjuvant approach to standard antiemetics for CINV management in BC patients.

Acupoint stimulations are believed to be associated with different degrees of non-specific effects which are usually referred to as placebo effects [59]. To distinguish the specific treatment effects of an intervention from its non-specific (placebo) effects, a placebo comparison is usually designed [60]. The study findings indicated a superiority of both the true AA and sham AA to standard care in alleviating CINV. However, statistically significant differences were only demonstrated between the true AA group and the standard care group, although clinically significant differences were found in some comparisons between the sham AA group and the standard care group. A further comparison between the true AA and the sham AA revealed relatively favourable CINV outcomes in the true AA group although none of the differences achieved statistical significance. The antiemetic effects of AA were therefore deemed as a mixture of specific treatment effects and placebo effects, given that both the true and sham AA were effective in alleviating CINV with relatively stronger antiemetic effects identified by using the true AA. Given the clinical significance identified in a few CINV outcomes between the sham AA group and the standard care group, the placebo effects of AA could be very large and clinically important to BC patients and clinicians. Our findings regarding the placebo effects of AA is consistent with a recent systematic review that focused on sham acupressure, which concluded that both true acupressure and sham acupressure are superior to conventional care, and the effects of true acupressure are generally larger when compared with the sham modalities [59].

Research evidence [5] has implicated that improved management of CINV during the first chemotherapy cycle may help reduce anticipatory nausea in the following cycle. However, our study did not reveal any positive effects of AA on anticipatory CINV. The very limited study sample included in the anticipatory CINV analysis could be the major reason for contributing to such insignificant outcomes. Burish and Carey (cited in [61]) indicated that the complex mechanism in the development of anticipatory emesis involving both neurological to psychophysiological factors can make the management of anticipatory CINV a challenging task. Complex interventions focusing on behavior changes might contribute to some potentially beneficial effects for alleviating anticipatory nausea and vomiting [61]. However, the AA treatment used in this study was not primarily designed for behavior changes, which might partially lead to the insignificant results on AA for anticipatory CINV management.

Participants’ QoL deteriorated across the first chemotherapy cycle, which implicated that the cancer treatment and related unpleasant symptoms gradually decreased the participants’ functional status. The onset of the CINV symptoms during the delayed phase may partially contribute to the deterioration and insignificant changes of QoL status among groups, as the ongoing experiences of CINV distress with a longer period have been linked with more deteriorated QoL [62]. In addition, the concept of QoL in cancer patients consists of different dimensions— physical, emotional, social, and functional [63]; thus, changes of QoL status may be more sensitive to those complex interventions with multidimensional components aimed at physical and psychosocial support. The AA utilized in this study mainly focuses on physical symptom alleviation but not psychological support, which could potentially contribute to the insignificant results in participants’ QoL status.

The confounding effects of the baseline CINV risk factors were minimal and did not change the between-group mean differences and related effect sizes of the antiemetic effect analysis of AA on CINV. However, future studies must consider relevant strategies to manage the possible confounders to the utmost extent at the research design stage. A restricted randomization approach could be applied to ensure the balance of patients’ CINV risk factors and other known risk factors associated with CINV during the randomization procedure.

This study has some strengths. The three-group design by including a sham intervention group and a no-intervention group enabled a further analysis to distinguish the specific treatment effects of AA from its placebo effects, as well as to size the placebo effects of AA for CINV management. Development of the AA intervention was based on a comprehensive analysis of AA-related theories, systematic review and clinical trial evidence, practice standards, CINV characteristics, and an expert panel’s consensus [29], which potentially contributed to an evidence-based AA intervention that can be adopted in routine practice as a standard protocol for CINV management. The inclusion of clinical significance in the data analysis further extended the value of the study outcomes to both BC patients and clinicians to support their clinical decision-making. Participant adherence to the study intervention was identified to be excellent as most of the participants followed the study instructions to complete the intervention and outcome assessments, which can be partially attributed to the safety and convenience of the 5-day AA intervention and the quality assurance strategies that were enforced during the study process. However, the study also has some limitations. As mentioned earlier, the sample size of this preliminary RCT was relatively small and the sample size estimation was not power-based. Thus, some statistical analyses might be underpowered which indicate a risk of committing a type II error (an error of omission) in the data analysis. The small sample size inhibited a further subgroup analysis of participants’ CINV symptoms between different antiemetic protocols. The partial blinding design of this study without masking participants and outcome assessment in the standard care group might, more or less, bias the outcome measures. Data analysis results of this RCT can therefore only be interpreted as preliminary findings, and the definite effects of AA on CINV and QoL in BC patients need to be further explored in a future fully-powered main RCT. Assessment of nausea and vomiting symptoms was only conducted through patient-reported outcome measures which could be quite subjective sometimes despite that both the MAT and INVR are well-validated with satisfactory psychometric properties documented. The randomization procedure without any restrictions on known CINV risk factors is also a limitation, as the additional data analysis indicated that the statistically insignificant variations in the patients’ baseline CINV risk factors across groups led to some confounding effects in the causality analysis between the AA and the CINV outcomes, although such effects were deemed to be minimal.

## Conclusion

This study concluded preliminary research evidence that the use of AA plus standard antiemetic treatment and care was superior to the use of standard antiemetic treatment and care alone in managing CINV among BC patients receiving chemotherapy. The antiemetic effects of AA were identified to be more profound in improving acute CINV, particularly acute nausea. The antiemetic effects of AA were deemed to be a mixture of specific treatment effects and placebo effects, and the placebo effects were very large and even reached clinical significance.

## Data Availability

The datasets used during the current study are available from the corresponding author on reasonable request.

## Abbreviations

AA: Auricular acupressure
BC: Breast cancer
CINV: Chemotherapy-induced nausea and vomiting
CR: Complete response
CVI: Content Validity Index
FACT-B: Functional Assessment of Cancer Therapy-Breast
INVR: Index of Nausea, Vomiting, and Retching
ITT: Intention-to-Treat
LOCF: Last Observation Carried Forward
MAT: MASCC Antiemesis Tool

## References

[1] Rao KV, Faso A (2012). Chemotherapy-induced nausea and vomiting: Optimizing prevention and management. Am Health Drug Benefits.

[2] Escobar Y, Cajaraville G, Virizuela JA, Álvarez R, Muñoz A, Olariaga O, Martínez P (2015). Incidence of chemotherapy-induced nausea and vomiting with moderately emetogenic chemotherapy: ADVICE (Actual Data of Vomiting Incidence by Chemotherapy Evaluation) study. Support Care Cancer.

[3] Hsieh RK, Chan A, Kim HK, Yu S, Kim JG, Lee MA, Keefe DM (2015). Baseline patient characteristics, incidence of CINV, and physician perception of CINV incidence following moderately and highly emetogenic chemotherapy in Asia Pacific countries. Support Care Cancer.

[4] Kottschade L, Novotny P, Lyss A, Mazurczak M, Loprinzi C, Barton D (2016). Chemotherapy-induced nausea and vomiting: Incidence and characteristics of persistent symptoms and future directions NCCTG N08C3 (Alliance). Support Care Cancer.

[5] Molassiotis A, Lee PH, Burke TA, Dicato M, Gascon P, Roila F, Aapro M (2016). Anticipatory nausea, risk factors, and its impact on chemotherapy-induced nausea and vomiting: Results from the Pan European Emesis Registry study. J Pain Symptom Manage.

[6] Rha SY, Park Y, Song SK, Lee CE, Lee J (2016). Controlling chemotherapy-induced nausea requires further improvement: Symptom experience and risk factors among Korean patients. Support Care Cancer.

[7] Olver I (2015). Assessing the burden and management of chemotherapy induced emesis in the Asia/Pacific region. Support Care Cancer.

[8] Farrell C, Brearley SG, Pilling M, Molassiotis A (2013). The impact of chemotherapy-related nausea on patients’ nutritional status, psychological distress and quality of life. Support Care Cancer.

[9] Hamadani M, Chaudhary L, Awan FT, Khan JK, Kojouri K, Ozer H, Tfayli A (2007). Management of platinum-based chemotherapy-induced acute nausea and vomiting: Is there a superior serotonin receptor antagonist?. J Oncol Pharm Pract.

[10] Osoba D, Zee B, Warr D, Latreille J, Kaizer L, Pater J (1997). Effect of postchemotherapy nausea and vomiting on health-related quality of life. Support Care Cancer.

[11] Schnell FM (2003). Chemotherapy-induced nausea and vomiting: The importance of acute antiemetic control. Oncologist.

[12] American Cancer Society. (2016). Nausea and vomiting: Chemotherapy-related nausea and vomiting. Retrieved from https://old.cancer.org/acs/groups/cid/documents/webcontent/003200-pdf.pdf.

[13] Chan VT, Yeo W (2011). Antiemetic therapy options for chemotherapy-induced nausea and vomiting in breast cancer patients. Breast Cancer: Targets and Therapy.

[14] Inrhaoun H, Kullmann T, Elghissassi I, Mrabti H, Errihani H (2012). Treatment of chemotherapy-induced nausea and vomiting. J Gastrointest Cancer.

[15] Lohr L (2008). Chemotherapy-induced nausea and vomiting. Cancer J.

[16] Thompson N (2012). Optimizing treatment outcomes in patients at risk for chemotherapy-induced nausea and vomiting. Clin J Oncol Nurs.

[17] Aapro, M., Gralla, R. J., Herrstedt, J., Molassiotis, A., & Roila, F. (2016). MASCC/ESMO Antiemetic Guideline 2016. MASCC. Retrieved from http://www.mascc.org/assets/Guidelines-Tools/mascc_antiemetic_guidelines_english_2016_v.1.2.pdf. 10.1007/s00520-016-3324-x 27501964

[18] Bray F, Ferlay J, Soerjomataram I, Siegel R L, Torre L A, Jemal A (2018). Global cancer statistics 2018: GLOBOCAN estimates of incidence and mortality worldwide for 36 cancers in 185 countries. CA: Cancer J Clin.

[19] Razvi Y, Chan S, McFarlane T, McKenzie E, Zaki P, DeAngelis C, Jerzak KJ (2019). ASCO, NCCN, MASCC/ESMO: a comparison of antiemetic guidelines for the treatment of chemotherapy-induced nausea and vomiting in adult patients. Support Care Cancer.

[20] Oncology Nursing Society [ONS]. (December 2019). ONS PEP: Chemotherapy-induced nausea and vomiting—Adult. Retrieved from https://www.ons.org/pep/chemotherapy-induced-nausea-and-vomiting-adult.

[21] Lee MS, Shin BC, Suen LKP, Park TY, Ernst E (2008). Auricular acupuncture for insomnia: A systematic review. Int J Clin Pract.

[22] Li MK, Lee TD, Suen KL (2014). Complementary effects of auricular acupressure in relieving constipation symptoms and promoting disease-specific health-related quality of life: A randomized placebo-controlled trial. Complement Ther Med.

[23] Shin J, Park H (2016). Effects of auricular acupressure on constipation in patients with breast cancer receiving chemotherapy: A randomized control trial. West J Nurs Res.

[24] Zhao HJ, Tan JY, Wang T, Jin L (2015). Auricular therapy for chronic pain management in adults: A synthesis of evidence. Complement Ther Clin Pract.

[25] Bai XH (1994). Chinese auricular therapy.

[26] Suen LK, Wong TK, Leung AW (2001). Is there a place for auricular therapy in the realm of nursing?. Complement Ther Nurs Midwifery.

[27] Tan, J. Y., Molassiotis, A., Wang, T., & Suen, L. K. (2014). Current evidence on auricular therapy for chemotherapy-induced nausea and vomiting in cancer patients: A systematic review of randomized controlled trials. Evidence-Based Complementary and Alternative Medicine, 2014, Article ID 430796, 18 pages, 10.1155/2014/430796. 10.1155/2014/430796 PMC4261635 25525445

[28] Tan J (2017). Effects of auricular acupressure on chemotherapy-induced nausea and vomiting in breast cancer patients: a preliminary randomized controlled trial. Doctoral dissertation,.

[29] Tan JY, Liu J, Suen LK, Molassiotis A, Wang T (2020). Development and validation of an evidence-based auricular acupressure intervention for managing chemotherapy-induced nausea and vomiting in breast cancer patients. Complement Ther Med.

[30] Chief Scientist Office. (2014). Development, pilot and feasibility studies. Retrieved from www.cso.scot.nhs.uk/wp-content/.../Development-pilot-and-feasibility-studies.docx.

[31] Hertzog MA (2008). Considerations in determining sample size for pilot studies. Res Nurs Health.

[32] Lancaster GA, Dodd S, Williamson PR (2004). Design and analysis of pilot studies: Recommendations for good practice. J Eval Clin Pract.

[33] Craig P, Dieppe P, Macintyre S, Michie S, Nazareth I, Petticrew M (2008). Developing and evaluating complex interventions: The new Medical Research Council guidance. BMJ.

[34] Molassiotis A, Coventry PA, Stricker CT, Clements C, Eaby B, Velders L, Gralla RJ (2007). Validation and psychometric assessment of a short clinical scale to measure chemotherapy-induced nausea and vomiting: The MASCC antiemesis tool. J Pain Symptom Manage.

[35] Tan JY, Suen LK, Molassiotis A (2016). Psychometric assessment of the Chinese version of the MASCC Antiemesis Tool (MAT) for measuring chemotherapy-induced nausea and vomiting. Support Care Cancer.

[36] Brearley SG, Clements CV, Molassiotis A (2008). A review of patient self-report tools for chemotherapy-induced nausea and vomiting. Support Care Cancer.

[37] Rhodes VA, McDaniel RW (1999). The Index of Nausea, Vomiting, and Retching: A new format of the Index of Nausea and Vomiting. Oncol Nurs Forum.

[38] Rhodes VA, McDaniel RW (2001). Nausea, vomiting, and retching: Complex problems in palliative care. CA: A Cancer Journal for Clinicians.

[39] Fu MR, Rhodes V, Xu B (2002). The Chinese translation of the index of nausea, vomiting, and retching. Cancer Nurs.

[40] Brady MJ, Cella DF, Mo F, Bonomi AE, Tulsky DS, Lloyd SR, Shiomoto G (1997). Reliability and validity of the Functional Assessment of Cancer Therapy-Breast quality-of-life instrument. J Clin Oncol.

[41] Webster K, Cella D, Yost K (2003). The Functional Assessment of Chronic Illness Therapy (FACIT) measurement system: Properties, applications, and interpretation. Health Qual Life Outcomes.

[42] Ng R, Lee CF, Wong NS, Luo N, Yap YS, Lo SK, Cheung YB (2012). Measurement properties of the English and Chinese versions of the Functional Assessment of Cancer Therapy-Breast (FACT-B) in Asian breast cancer patients. Breast Cancer Res Treat.

[43] Wan C, Zhang D, Yang Z, Tu X, Tang W, Feng C, Tang X (2007). Validation of the simplified Chinese version of the FACT-B for measuring quality of life for patients with breast cancer. Breast Cancer Res Treat.

[44] World Health Organization. (2005). The use of the WHO-UMC system for standardized case causality assessment. Uppsala: The Uppsala Monitoring Centre. Retrieved from http://whoumc.org/Graphics/24734.pdf.

[45] Moradian S (2013). Management of chemotherapy-induced nausea and vomiting: A pilot randomised controlled trial using Nevasic audio programme (Unpublished doctoral dissertation).

[46] Popovic M, Warr DG, DeAngelis C, Tsao M, Chan KK, Poon M, Chow E (2014). Efficacy and safety of palonosetron for the prophylaxis of chemotherapy-induced nausea and vomiting (CINV): A systematic review and meta-analysis of randomized controlled trials. Support Care Cancer.

[47] Saito M, Aogi K, Sekine I, Yoshizawa H, Yanagita Y, Sakai H, Mitsuhashi S (2009). Palonosetron plus dexamethasone versus granisetron plus dexamethasone for prevention of nausea and vomiting during chemotherapy: A double-blind, double-dummy, randomised, comparative phase III trial. Lancet Oncol.

[48] Liao SY, Meskin A (2015). Aesthetic adjectives: Experimental semantics and context-sensitivity. Philos Phenomenol Res.

[49] McHugh ML (2013). The chi-square test of independence. Biochemia Medica.

[50] Coolican H (2014). Research methods and statistics in psychology.

[51] Simoens VL, Tervaniemi M (2013). Auditory short-term memory activation during score reading. PloS One.

[52] Fritz CO, Morris PE, Richler JJ (2012). Effect size estimates: Current use, calculations, and interpretation. J Exp Psychol Gen.

[53] Cohen J (1988). Statistical power analysis for the behavioral sciences.

[54] Medical Research Council Cognition and Brain Sciences Unit. (July 2016). Rules of thumb on magnitudes of effect sizes. Retrieved from http://imaging.mrc-cbu.cam.ac.uk/statswiki/FAQ/effectSize.

[55] Buczkowski K, Basinska MA, Ratajska A, Lewandowska K, Luszkiewicz D, Sieminska A (2017). Smoking status and the five-factor model of personality: Results of a cross-sectional study conducted in Poland. Int J Environ Res Public Health.

[56] Dempster M (2011). A research guide for health and clinical psychology.

[57] Guyatt, G. H., Osoba, D., Wu, A. W., Wyrwich, K. W., Norman, G. R., & Clinical Significance Consensus Meeting Group (2002). Methods to explain the clinical significance of health status measures. Mayo Clin Proc.

[58] Man-Son-Hing M, Laupacis A, O’Rourke K, Molnar FJ, Mahon J, Chan KB, Wells G (2002). Determination of the clinical importance of study results. J Gen Intern Med.

[59] Tan JY, Suen LK, Wang T, Molassiotis A (2015). Sham acupressure controls used in randomized controlled trials: A systematic review and critique. PloS One.

[60] Dincer F, Linde K (2003). Sham interventions in randomized clinical trials of acupuncture—A review. Complement Ther Med.

[61] Roscoe JA, Morrow GR, Aapro MS, Molassiotis A, Olver I (2011). Anticipatory nausea and vomiting. Support Care Cancer.

[62] Fernandez-Ortega P, Caloto MT, Chirveches E, Marquilles R, San Francisco J, Quesada A, Nocea G (2012). Chemotherapy-induced nausea and vomiting in clinical practice: Impact on patients’ quality of life. Support Care Cancer.

[63] Holzner B, Bode RK, Hahn EA, Cella D, Kopp M, Sperner-Unterweger B, Kemmler G (2006). Equating EORTC QLQ-C30 and FACT-G scores and its use in oncological research. Eur J Cancer.

